# Radiation dose levels in chest computed tomography scans of coronavirus disease 2019 pneumonia: A survey of 2119 patients in Chongqing, southwest China

**DOI:** 10.1097/MD.0000000000026692

**Published:** 2021-08-06

**Authors:** Yang Zhou, Yineng Zheng, Yun Wen, Xin Dai, Wengang Liu, Qihui Gong, Chaoqiong Huang, Fajin Lv, Jiahui Wu

**Affiliations:** a Department of Radiology, The First Affiliated Hospital of Chongqing Medical University, Chongqing, PR China; b Department of Radiology, Chongqing University Three Gorges Hospital, Chongqing, PR China; c Department of Radiology, Chongqing Public Health Medical Center, Chongqing , PR China; d Department of Radiology, Yongchuan Hospital of Chongqing Medical University, Chongqing, PR China.

**Keywords:** computed tomography, coronavirus disease 2019 pneumonia, effective dose, radiation exposure

## Abstract

To investigate computed tomography (CT) diagnostic reference levels for coronavirus disease 2019 (COVID-19) pneumonia by collecting radiation exposure parameters of the most performed chest CT examinations and emphasize the necessity of low-dose CT in COVID-19 and its significance in radioprotection.

The survey collected RIS data from 2119 chest CT examinations for 550 COVID-19 patients performed in 92 hospitals from January 23, 2020 to May 1, 2020. Dose data such as volume computed tomography dose index, dose-length product, and effective dose (ED) were recorded and analyzed. The radiation dose levels in different hospitals have been compared, and average ED and cumulative ED have been studied.

The median dose-length product, volume computed tomography dose index, and ED measurements were 325.2 mGy cm with a range of 6.79 to 1098 mGy cm, 9.68 mGy with a range of 0.62 to 33.80 mGy, and 4.55 mSv with a range of 0.11 to 15.37 mSv for COVID-19 CT scanning protocols in Chongqing, China. The distribution of all observed EDs of radiation received by per patient undergoing CT protocols during hospitalization yielded a median cumulative ED of 17.34 mSv (range, 2.05–53.39 mSv) in the detection and management of COVID-19 patients. The average number of CT scan times for each patient was 4.0 ± 2.0, and the average time interval between 2 CT scans was 7.0 ± 5.0 days. The average cumulative ED of chest CT examinations for COVID-19 patients in Chongqing, China greatly exceeded public limit and the annual dose limit of occupational exposure in a short period.

For patients with known or suspected COVID-19, a chest CT should be performed on the principle of rapid-scan, low-dose, single-phase protocol instead of routine chest CT protocol to minimize radiation doses and motion artifacts.

## 1 Introduction

The outbreak of coronavirus disease 2019 (COVID-19) caused by the novel, pathogenic SARS-coronavirus 2 and started December 2019, has rapidly spread across >200 countries.^[1]^ Pneumonia is the one of most common manifestations in COVID-19 that invades and attacks respiratory system, where can appear in the chest computed tomography (CT) imaging of COVID-19 patients.

Chest CT imaging plays an important role in detecting COVID-19 before receiving the reverse transcription-polymerase chain reaction (RT-PCR) nucleic acid testing, and is majorly helpful in screening and management for suspected COVD-19 patients with epidemiologic features compatible with COVID-19 infection particularly but negative RT-PCR tests.^[2,3]^ Therefore, it can be considered a useful test for relieving quickly difficult situations, especially in centers that laboratory tests may not be easily accessible. due to the lack of kits, delay waiting for the results, as well as false negative cases.^[4]^

Moreover, on the basis of the 7th trial version of diagnosis and treatment of COVID-19,^[5]^ the hospitalized patients should receive follow-up CT to evaluate the changes of pneumonia or quantify the inflammation absorption and dissipation of the lungs in chest CT images during the follow-up scanning, and the discharged patients need also close medical observation by follow-up CT.^[6,7]^ Actually, some suspected patients may receive one or more CT examinations to ensure they are healthy within a short period of time.^[4,8]^ Therefore, medical radiation exposure of multiple CT should be more concerned,^[9]^ especially for special community such as children and pregnant women. But until now, the average accumulation dose per COVID-19 patient within hospitalization has not been well known.

The aim of this study was to investigate the current radiation dose levels for chest CT examinations in COVID-19 patients by collecting the primary dosimetry metrics such as dose-length product (DLP), volume computed tomography dose index (CTDI_vol_), and effective dose (ED) for each examination and calculating the cumulative ED for each patient, and comparing the differences of those in different designated hospitals admitting COVID-19 patients throughout Chongqing, China. Based on this data, to make radiological technicians know the real radiation exposure levels of COVID-19 patients and the necessity of low-dose CT in COVID-19 and its significance in radioprotection.

## 2 Materials and methods

### 2.1 Participants

This study was subject to approval by the Ethics Committee of our hospital. Signed informed consent was exempted due to the retrospective nature of the study. This survey collected the detailed protocols of 2119 non-contrast chest CT examinations from 550 patients with COVID-19 in 92 hospitals consisting of 3 designated hospitals and 89 first-visited hospitals of those throughout Chongqing Municipality. The data were collected from January 23, 2020 to May 1, 2020. Inclusion criteria were as follows: patients with laboratory-confirmed SARS-coronavirus 2 infection by RT-PCR in Chongqing who underwent both chest CT scan and RT-PCR examinations. Exclusion criteria: the CT image quality of the patients did not meet the need of diagnosis for radiologist. Exclusion criteria: failure to provide complete dose reports or incomplete questionnaires impacted on statistical results.

### 2.2 Data collected

The data collected from each hospital included the scale of hospital, the information and the conditions of patients, chest CT protocols employed, and the CTDI_vol_ and DLP values displayed on dose report of CT scanner during each non-contrast chest CT scan. Examination times, time interval between 2 successive CT scans, and the average value between the minimum and maximum values of CTDI_vol_, DLP, and ED for each patient were calculated, where ED was calculated using a conversion factor (*k*) of 0.0145 mSv × mGy^−1^ × cm^−1^.^[10]^ The cumulative ED can be obtained by summing all observed EDs of radiation received by per patient undergoing CT protocols during hospitalization.

### 2.3 Statistical analysis

The statistical analysis and computations were performed using R software (version 3.5.2, R Foundation for Statistical Computing, Vienna, Austria, https://www.r-project.org/). Continuous data were presented as mean ± standard deviation and categorical data were reported as counts and the percentage of the total. The normality and variance homogeneity of continuous variables were evaluated using the Shapiro-Wilk test and Levene test, respectively. Significance analyses of normally distributed and quantitative data with equal variance between groups were performed using unpaired or paired Student *t* test, while Mann–Whitney *U* test or Wilcoxon signed-rank test was used for the quantitative data that did not follow a normal distribution. Group comparisons of categorical data were performed with the use of Fisher exact test or chi-square test. The threshold of statistical significance was set to a 2-sided *α* of <0.05.

## 3 Results

**Figure 1:**
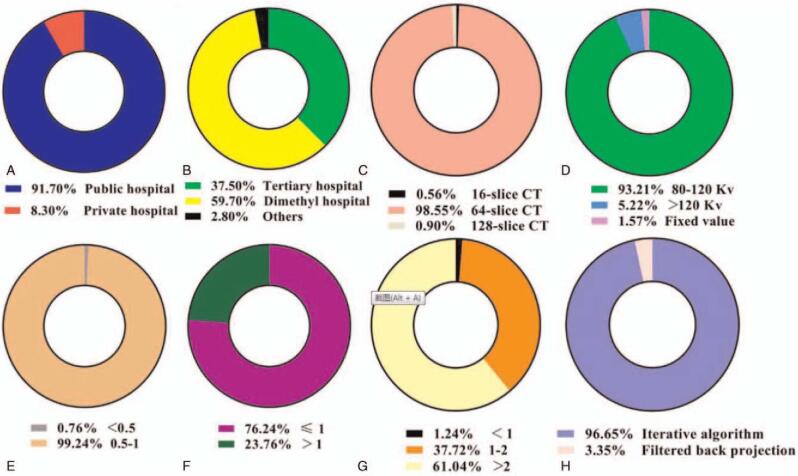
The proportion of basic information of the chest CT scanning protocols for COVID-19 in 92 hospitals including (A) hospital nature, (B) hospital level, (C) multi-slice capability, (D) tube voltage, (E) rotate speed, (F) pitch, (G) slice thickness, and (H) types of reconstruction algorithms. COVID-19 = coronavirus disease 2019, CT = computed tomography.

This survey collected the detailed protocols of 2119 non-contrast chest CT examinations from 550 patients with COVID-19 in 92 hospitals consisting of 3 designated hospitals and 89 first-visited hospitals of those. The demographic information of registered patients was presented in Table 1. The basic information and the chest CT scanning protocols for COVID-19 of 92 hospitals including hospital level, multi-slice capability of CT scanners, tube voltage, rotate speed, pitch, imaged slice thickness, and the type of reconstruction algorithms were shown in Fig. 1, of which 504 are public and 46 are private, and the proportion of tertiary hospitals reached 37.5%. All scanners surveyed had multi-slice capability ranging from 2 to 256 slices (Fig. 1), of which 16-slice or 64-slice CT scanners were the most common with tube voltages (range, 80–120 kV), rotate speeds (range, 0.5–1.0 r/min), pitch (range, 0.7–1.5), slice thickness (range, 2–5 mm) using iterative reconstruction algorithm.

**Table 1:** The demographic information and clinical characteristics.

Characteristics	All patients (N = 550)
Age, y	47 (16)
Sex	
Female	254 (46.18%)
Male	296 (53.82%)
Radiological examination	
Only DR	0
Only CT	465 (84.5%)
CT and DR	85 (15.5%)

**Figure 2:**
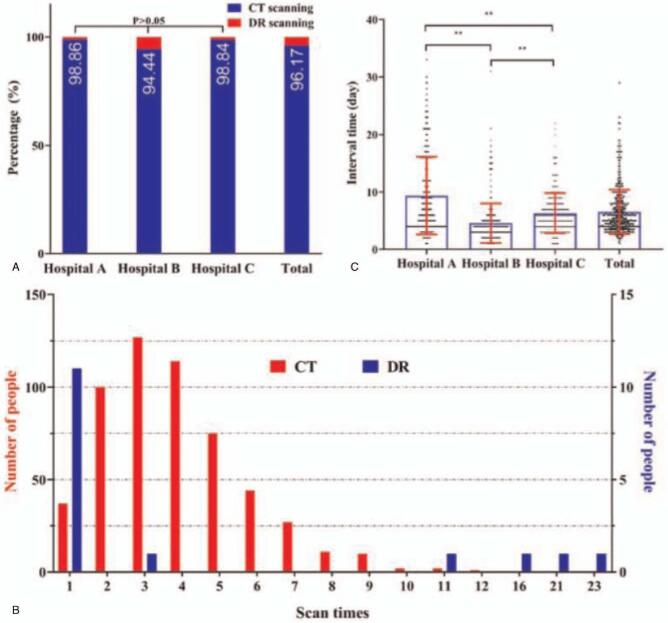
The basic information about radiological examination for COVID-19. (A) The proportion of imaging techniques for CT and DR in 3 designated hospitals and the total hospitals. (B) The frequency distribution of CT and DR scanning times. (C) The interactive bar graph and scatter plot applications for showing the time intervals between 2 successive CT scans for COVID-19 patients. COVID-19 = coronavirus disease 2019, CT = computed tomography, DR = digital radiography.

The proportion of CT and digital radiography (DR) scans for detection and management of COVID-19 in 3 designated hospitals of Chongqing, southwest China was shown in Fig. 2A. It indicated that chest CT is the main radiological technique. The frequency distribution of CT and DR scanning times for the 550 COVID-19 patients in Fig. 2B shown that the most patients received the number of CT scans ranges from 2 to 6 during their hospitalization. The time interval between 2 successive CT scans for each COVID-19 patient in these designated hospitals was illustrated in Fig. 2C. The average number of CT scan times for each patient was 4.0 ± 2.0, and the average time interval between 2 CT scans was 7.0 ± 5.0 days.

**Figure 3:**
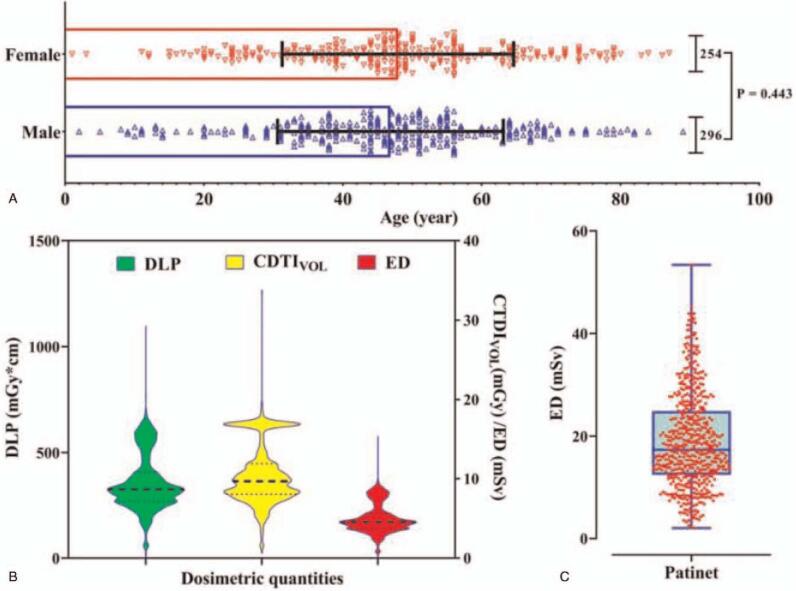
The statistical result of 2119 non-enhanced chest CT scans. (A) the age and sex distribution of 550 COVID-19 patients. (B) The violin plot of the radiation exposure parameters such as DLP, CDTI_vol_, and ED. (C) The violin plot of cumulative ED per patient during hospitalization. COVID-19 = coronavirus disease 2019, CT = computed tomography, CTDIvol = volume computed tomography dose index, DLP = dose-length product, ED = effective dose.

The dosimetry quantities of the surveyed 2119 CT examinations for 550 COVID-19 patients were shown in Fig. 3, of which 254 women and 296 men were included without significant difference in age (Fig. 3A). The median DLP (325.2 mGy cm; range, 6.79–1098 mGy cm), CTDI_vol_ (9.68 mGy; range, 0.62–33.80 mGy), and ED (4.55 mSv; range, 0.11–15.37 mSv) for COVID-19 CT scanning protocols in Chongqing, China were summarized (Table 2), and the corresponding distributions of radiation dose descriptors were shown in Fig. 3B. The distribution of all observed EDs of radiation received by per patient undergoing CT protocols during hospitalization yielded a median cumulative effective dose of 17.34 mSv (range, 2.05–53.39 mSv) for all patients in the detection and management of COVID-19 (Fig. 3C and Table 2).

**Table 2:** Descriptive statistics of the radiation dose descriptors of the surveyed 2119 CT examinations for 550 COVID-2019 patients in Chongqing, China.

	Median	Range	25th to 75th percentile	Mean	Standard deviation
CT examinations (N = 2119)					
DLP, mGy cm	325.2	6.79–1098	268.0–405.6	355.22	130.51
CTDI_VOL_, mGy	9.680	0.62–33.80	8.04–11.92	10.48	3.79
ED, mSv	4.55	0.11–15.26	3.75–5.68	4.976	1.824
COVID-2019 patient (N = 550)					
Cumulative ED, mSv	17.34	2.058–53.39	12.43–24.89	19.07	9.25
DLP_max-min_, mGy cm	113.4	0.20–659.2	47.24–254.3	159.43	135.50
CTDI_VOLmax-min_, mGy	2.25	0.32–189.3	0.92–7.10	4.382	9.17
ED_max-min_, mSv	1.58	0.003–9.23	0.66–3.56	2.23	1.89

**Figure 4:**
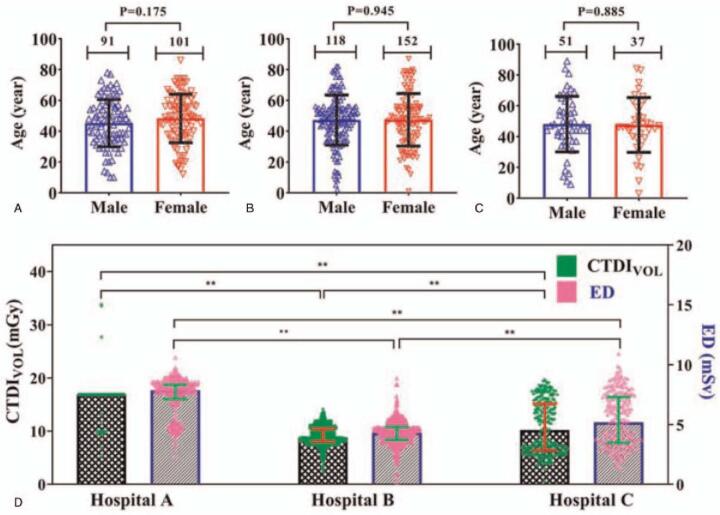
The comparison of radiation exposure parameters among 3 designated hospitals. (A)–(C) The age and sex distribution of COVID-19 patients in Hospital A, B, and C. The interactive bar graph and scatter plot applications for showing the levels of CDTI_vol_ and ED per CT examination for the 3 designated hospitals. COVID-19 = coronavirus disease 2019, CT = computed tomography, CTDIvol = volume computed tomography dose index, ED = effective dose.

**Figure 5:**
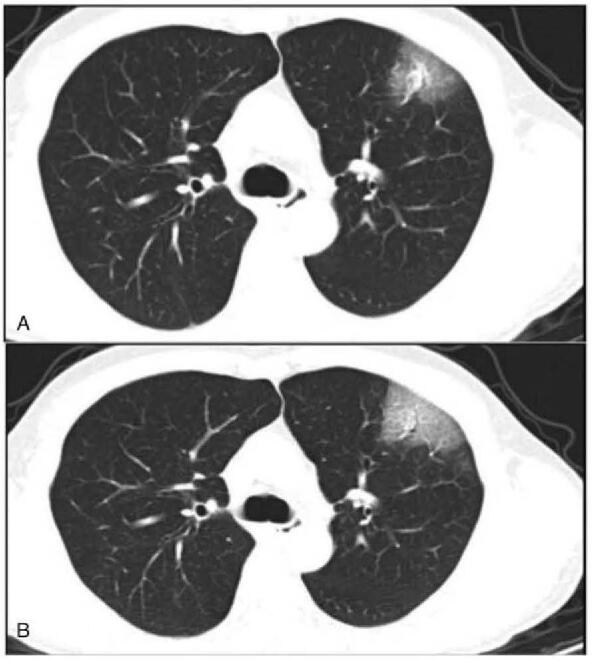
A 49-year-old man received 2 chest CT scans by the same modality within 3 days. (A) Image obtained in the first time CT scan with CTDI_vol_, DLP, ED were 5.16 mGy, 200.5 mGy cm, and 2.81 mSv. (B) Image obtained in the second time CT scan with CTDI_vol_, DLP, ED were 1.67 mGy, 68.46 mGy cm, 0.96 mSv.

The comparison results of surveyed data from the first-visited hospital and 3 designated hospitals (tertiary hospitals) were shown in Tables 3 and 4. The sex (*P* = .285) and age (*P* = .374) of COVID-19 patients from these 3 hospitals were no statistically significant difference (Fig. 4A–C). Our survey found that the CTDI_vol_, DLP, and ED have obvious significant difference among the 3 designated hospitals (*P* < .001) and between first-visited and treatment hospitals (*P* < .001). The COVID-19 patients in Hospital A received a significantly higher effective dose per CT examination (median 7.85 mSv; range, 2.35–10.60 mSv) than did COVID-19 patients in Hospital B (median, 4.31 mSv; range, 0.11–8.86 mSv) and those in Hospital C (median, 5.18 mSv; range, 0.32–10.91 mSv) (*P* < .001, Fig. 4D). The mean values of CTDI_vol_, DLP, and ED ranges were 4.38 ± 9.17 mGy, 159.43 ± 135.50 mGy cm, and 2.23 ± 1.89 mSv in multiple CT scans for a patient, particularly the maximum range of ED up to 9.23 mSv for a certain patient. Figure 5 shown a patient received 2 chest CT examinations by a 64-slice detector CT in a period, where the ED of the later scanning were 50% lower than that of the former scanning, while the image quality of both images meets the diagnostic criteria. These findings support the recommendation of low-dose CT in the detection and management of COVID-19.

**Table 3:** The compassion of radiation dose exposure parameters between the first-visited and designated hospitals.

	First-visited hospital	Designated hospital	*P* value
CT scanning times	N = 224	N = 1895	
CTDI_VOL_, mGy	8.91 (7.31–10.81)	9.78 (8.47–11.02)	.067^a^
DLP, mGy cm	276.70 (240.98–335.37)	326.49 (298.92–369.68)	<.001^a^
ED (*K* = 0.014), mSv	3.87 (3.37–4.70)	4.57 (4.18–5.18)	.008^a^

**Table 4:** The compassion of radiation dose exposure parameters among 3 designated hospitals.

	Hospital A	Hospital B	Hospital C	*P* value
CT scanning times	N = 383	N = 1241	N = 271	
CTDI_VOL_, mGy	16.9 (16.9–16.9)^∗#^	8.78 (8.04–10.45) ^∗†^	10.23 (6.4–15.17)^#†^	<.001^a^
DLP, mGy cm	560.7 (509.9–594.6)^∗#^	307.3 (266.1–342.6)^∗†^	370.0 (248.5–521.0)^#†^	<.001^a^
ED, mSv	7.85 (7.14–8.32)^∗#^	4.30 (3.72–4.79)^∗†^	5.18 (3.47–7.29)^#†^	<.001^a^

## 4 Discussion

CT imaging plays an important role in the diagnosis and management of COVID-19, especially in the early screening of the disease as well as follow up, where throat swab tests may be false-negative due to a low viral load.^[11,3]^ In the 7th version of COVID-19 diagnosis and treatment handbook of the People's Republic of China,^[5]^ CT was considered a major procedure for disease detection before the RT-PCR tests,^[12]^ and moreover COVID-19 patients should receive follow-up CT to monitor the changes of pneumonia for disease progression management during hospitalization.^[13,14]^ In clinical, the patients with COVID-19 received 2 to 5 chest CT examinations within a month.^[15]^ In certain an institution, the high numbers of DR on some patients were found. Due to the lack of CT in primary medical institutions, the rapid progression of COVID-19 disease, only a few of patients suffered from repeated DR examinations. Medical radiation exposure is therefore a concern, and radiation dose management for special patient population including infants, children, young individuals, and pregnant woman is particularly important.

Our survey has covered almost all hospitals that received and treated more than 95% admitted patients with COVID-19 pneumonia in Chongqing, southwest China, including 2119 chest CT imaging from 550 patients. The DLP in the designated hospitals (A, B, and C) is higher than that in the first-visited hospital, and the patients received effective dose seems higher in the designated hospitals, probably because the suspected COVID-19 patients were screened with low-dose CT in the first-visited hospital, while for the confirmed cases conventional CT were used to provide clear images of lung. The 3 major hospitals A, B, and C were the designated hospitals for receiving COVID-19. Once the patients were diagnosed with COVID-19, they will be transferred to the designated hospital for others, so this study mainly discusses the difference of dose level among different COVID-19 patients and among 3 major hospitals. The median DLP, CTDIvol, and ED measurements were 325.2 mGy cm with a range of 6.79 to 1098 mGy cm, 9.68 mGy with a range of 0.62 to 33.80 mGy, and 4.55 mSv with a range of 0.11 to 15.37 mSv for COVID-19 CT scanning protocols in Chongqing, China. The investigation indicates that the CT scanner type, chest CT protocols, and radiation doses in COVID-19 patients varied greatly within the same and between different medical institutions, and <1.13% scans reported median CTDIvol <3 mGy. Considering our results, the radiation risk of COVID-19 patients undergoing standard chest CT scans does not appear to be negligible, and therefore the recommendation of low-dose CT in the screening, diagnosis, and management of COVID-19 is necessary.

Some studies have recommended the use of single-phase, low-dose, and non-contrast chest CT protocols for COVID-19 patients.^[16,17]^ Kang et al^[15]^ implemented a low-dose CT scan protocol at ≤100 kV, 0.6-second exposure time and low tube current with tin filter and iterative reconstruction algorithm with a CTDIvol of 0.39 mGy (Siemens Healthcare). Homayounieh et al^[18]^ reported almost 30% of COVID-19 patients underwent 2 to 8 chest CT examinations within a month and the variation of dose descriptors was obviously prominent in median CTDIvol and DLP. This is consistent with our findings. In Homayounieh study, CTDIvol and DLP are the median from each healthcare site, so Lee^[19]^ considered the actual range of the dose descriptors all over the world must be considerably larger. Our study surveyed the scan protocol and dose descriptors for each patient at the individual level. Up to our knowledge, this is the first study to focus on the individual level-based investigation of medical radiation exposure, CT scan protocols, and radiation dose levels in COVID-19 patients from the medical centers of western China, while this study demonstrates that current CT dose levels are well above previously recommended values (3 mGy). These findings strongly recommend that low-dose chest CT scan protocols must be adopted, if necessary, no matter for screening the patients with suspected COVID-19 or monitoring progression and follow-up, to reduce the risk of cancer caused by high radiation doses.

The International Commission on Radiological Protection introduced the concept of diagnostic reference levels (DRLs) in 1996,^[20]^ American College of Radiology (ACR) issued the DRL of chest CT without contrast is 12 mGy in CTDI_vol_ in 2018,^[21]^ and ACR suggest chest low-dose CT protocol must have a CTDI_vol_ of ≤3 mGy.^[22]^ Of the 2119 CT scans, the average CTDI_vol_ was 10.57 ± 5.53 mGy where the radiation dose level may close to the DRL of Chest non-contrast CT proposed by ACR, but the average accumulate DLP and ED of the multiple scan were 1361.88 ± 663.86 mGy cm and 19.07 ± 9.26 mSv for a patient, and especially some patient received 53.39 mSv radiation within a short period of time. The total effective radiation dose greatly exceeded public limit of 1 mSv in all patients and 39.45% of them exceeded the annual dose equivalent limit, 20 mSv, of occupational exposure in a short team.^[23]^ All the CT images we received meet the diagnostic criteria of COVID-19. Although low-dose CT impairs the diagnostic image quality, especially in the mediastinal window, COVID-19 diagnosis was mostly dependent on the lung window. Moreover, with the deep understanding of COVID-19 image features, it can still be used for auxiliary diagnosis by radiologist. The most direct and effective methods for radiation dose reduction involve decreasing the tube voltage and tube current, the use of automatic tube current modulation, iterative reconstruction, and coarse pitch scanning. To balance the relationship between image quality and dose level to meet the needs of diagnosis, chest low-dose CT scan protocol with CTDIvol <3 mGy is recommended for the follow-up considering the accumulation dose within a short term, and the interval of imaging follow-up depends on the severity degree of disease.

Reasons resulting in such phenomena are as follows: The average chest CT re-examination times is 4.0 ± 3.0, and particularly few patients received multiple chest CT examinations >10 times. So far as I know, there are no evidence about how many times and how often did patient receive chest CT examinations reasonably. Rare application of low-dose CT was found in our survey, only 1.13% CTDI_vol_ <3 mGy. In this survey, 98.58% chest CT examinations were performed by separate 16-slice or 64-slice CT scanners for COVID-19 patients instead of high-end CT scanners (256-slice) that meet the needs of other complex examinations such as CT angiography. This helped to reduce the risk of in-hospital transmission and allowed to avoid time- and resource-consuming room decontamination procedures. Therefore, chest low-dose CT scanning protocol in the screening, diagnosis, and management of COVID-19 is highly recommended, especially in follow-up CT.

Several limitations also exist. First, our study enrolled a relatively small number of samples (550 patients from 92 institutions), and was also a retrospective survey on chest CT practices and protocols for the COVID-19 pneumonia patients in Chongqing, China, which may have generalization restrictions that do not apply to other districts. Second, the precision of the reported dose descriptors may be subject to errors, heterogeneity, and variations caused by manually recorded data from different medical institutions and CT equipments. Third, because the effective dose per DR examination cannot be counted, the cumulative dose of the high numbers of DR examinations was omitted and not compared with that of CT. The multicenter or international studies should be performed to objectively estimate the dose descriptor level in chest CT protocols for COVID-19 and optimize radiation dose management in future studies. Directly automatic acquisition of electronic dose records from PACS would be also sought to reduce manual error under investigation.

In summary, this study provides valuable information on chest CT radiation doses in COVID-19 patients. The survey results shown chest CT plays an important role in the diagnosis, progression monitoring, and treatment response of COVID-19 pneumonia in most medical institutions of Chongqing, China, while the scan protocols and radiation doses in COVID-19 patients presents wide variation across medical institutions. Although several optimization strategies on radiation dose management have already been proposed for CT protocols, there is little clinical use in COVID-19 pneumonia examination across Chongqing hospitals. For patients with known or suspected COVID-19, a chest CT should be performed on the principle of rapid-scan, low-dose, single-phase protocol instead of routine chest CT protocol to minimize radiation doses and motion artifacts.

## Author contributions

**Conceptualization:** Lead: Fajin Lv.

**Formal analysis:** Equal: Xin Dai, Qihui Gong, Lead: Yineng Zheng.

**Funding acquisition:** Lead: Fajin Lv.

**Investigation:** Equal: Yun Wen, Xin Dai, Wengang Liu, Qihui Gong, Lead: Fajin Lv.

**Methodology:** Equal: Xin Dai, Wengang Liu, Qihui Gong.

**Project administration:** Lead: Fajin Lv.

**Supervision:** Equal: Yun Wen, Lead: Fajin Lv.

**Validation:** Equal: Yun Wen, Xin Dai, Wengang Liu, Lead: Fajin Lv.

**Writing – original draft:** Yang Zhou, Yineng Zheng, Jiahui Wu.

**Writing – review & editing:** Yang Zhou, Yineng Zheng, Chaoqiong Huang, Jiahui Wu.
